# Unsupervised reduction of random noise in complex data by a row-specific, sorted principal component-guided method

**DOI:** 10.1186/1471-2105-9-508

**Published:** 2008-11-29

**Authors:** Joseph W Foley, Fumiaki Katagiri

**Affiliations:** 1 Department of Plant Biology, Microbial and Plant Genomics Institute, University of Minnesota, 1500 Gortner Ave., St. Paul, MN 55108, USA; 2 Current address: Graduate Genetics Program, Stanford University, Stanford, CA 94305, USA

## Abstract

**Background:**

Large biological data sets, such as expression profiles, benefit from reduction of random noise. Principal component (PC) analysis has been used for this purpose, but it tends to remove small features as well as random noise.

**Results:**

We interpreted the PCs as a mere signal-rich coordinate system and sorted the squared PC-coordinates of each row in descending order. The sorted squared PC-coordinates were compared with the distribution of the ordered squared random noise, and PC-coordinates for insignificant contributions were treated as random noise and nullified. The processed data were transformed back to the initial coordinates as noise-reduced data. To increase the sensitivity of signal capture and reduce the effects of stochastic noise, this procedure was applied to multiple small subsets of rows randomly sampled from a large data set, and the results corresponding to each row of the data set from multiple subsets were averaged. We call this procedure Row-specific, Sorted PRincipal component-guided Noise Reduction (RSPR-NR). Robust performance of RSPR-NR, measured by noise reduction and retention of small features, was demonstrated using simulated data sets. Furthermore, when applied to an actual expression profile data set, RSPR-NR preferentially increased the correlations between genes that share the same Gene Ontology terms, strongly suggesting reduction of random noise in the data set.

**Conclusion:**

RSPR-NR is a robust random noise reduction method that retains small features well. It should be useful in improving the quality of large biological data sets.

## Background

Biological data are likely to contain random noise. It may be possible to statistically identify and reduce such random noise, especially in large data sets. Principal component analysis (PCA), also known as singular value decomposition, has been used for the purpose of statistical reduction of random noise [1]. Small variances associated with higher-order principal components (PCs) are nullified as random noise. PCA has been used for analysis of large biological data sets, such as expression profile data [1,2]. Although PCA is useful for capturing major trends in data, it could result in loss of important information. A typical form of expression profile data is a matrix with thousands of rows (genes) and tens or hundreds of columns (biological samples). Large biological data sets usually have many small features as well as large ones. Small features consisting of small numbers of rows and columns do not contribute much to the overall variance of the data. Therefore, PCs associated with small features are among the high order PCs. Consequently, small but biologically important features may be removed as noise. It is inevitable that severe dimensionality reduction in a global space, such as that often applied using PCA, will result in loss of signal from truly high-dimensional data in which many dimensions are represented by small features.

Here we present a robust unsupervised method for reduction of random noise in large data sets, which we call Row-specific, Sorted PRincipal component-guided Noise Reduction (RSPR-NR). We applied the method to Arabidopsis Affymetrix microarray expression profile data collected in multiple experiments. The correlations between expression profiles of gene pairs that share Gene Ontology (GO) biological process and cellular component terms were significantly increased while those of gene pairs that do not share GO terms were decreased. RSPR-NR clearly exceeded the performance of PCA in this test using a real data set. Thus, RSPR-NR can facilitate gene discovery by reducing random noise while retaining small features in expression profile data.

## Results

### RSPR-NR algorithm

This noise reduction procedure can be applied to an *m *rows × *n *columns (*m *> *n*) data matrix, ***D***, in which the random noise is assumed to be normally distributed with a mean of zero. In general, we expect *m *and *n *to be in the range of thousands and 30–200, respectively. RSPR-NR was coded in R (ver. 2.5.1) [3]. The R script for RSPR-NR is freely available for non-commercial use at .

The core RSPR-NR procedure is as follows.

(1) PCA without centering or scaling was applied to ***D ***using the columns as the coordinates, to obtain the PC-coordinated data matrix ***P***. The values in each row of ***P ***are called the PC-coordinates of the data point (i.e., the row).

(2) In each *i*th row of ***P***, the PC-coordinates were squared and sorted in descending order, yielding the sorted squared PC-coordinate matrix, ***S***.

(3) For each element *s*_*ij *_in the *i*th row and the *j*th column in ***S***, the probability *p*_*ij *_= **P**(*x *> *s*_*ij*_), for which *s*_*ij *_is derived from the normal noise model, was determined using the *j*th order distribution of the squared random noise. The squared values of random values sampled from the standard normal distribution ***N***(0,1) assume the *χ*^2^-distribution with one degree of freedom. *c*(*x*; 1) and *C*(*x*; 1) denote the probability density function and the cumulative distribution function of the *χ*^2^-distribution with one degree of freedom, respectively. When *n *values sampled from *c*(*x*; 1) are put in decreasing order, the value in the *j*th rank is drawn from a distribution with probability density function

$$f_{j}(x)=\frac{n!}{(j-1)!(n-j)!}C{\left(x;1\right)}^{n-j}{\left(1-C(x;1)\right)}^{j-1}c(x;1),$$

and cumulative distribution function

*F*_*j*_(*x*) = *B*(*C*(*x*; 1); *n *- *j *+ 1, *j*),

where *B*(*y*; *u*, *w*) is the cumulative distribution function of the Beta distribution with parameters *u *and *w *[4].

To calculate *p*_*ij*_, the scaling factor *a*_*i *_for the *i*th row was determined as

$$a_{i}=\frac{\text{median}(F_{k}^{-1}(0.5)|k=1,2,\ldots ,n)}{\text{median}(s_{ik}|k=1,2,\ldots ,n)}$$

This scaling was based on the assumption that the median and the higher ranked columns in the *i*th row of ***S ***contain no signals but noise. Then

*p*_*ij *_= 1 - *F*_*j*_(*a*_*i*_*s*_*ij*_).

For practical efficiency, only *p*_*ij *_for $j\leq \left\lceil \frac{n}{2}\right\rceil$ were calculated because of this assumption. These *p*_*ij *_were corrected for Benjamini-Hochberg False Discovery Rate (FDR) [5] to obtain *q*_*ij*_. The remaining *q*_*ij *_for $j>\left\lceil \frac{n}{2}\right\rceil$ were set to 1.

(4) Noise-reduced data was generated. The elements in the PC-coordinate data matrix ***P ***corresponding to the *q*_*ij *_that were larger than the preset FDR were nullified as random noise to obtain a noise reduced matrix, ***P***_*nr*_. Note that the column positions of the corresponding elements in ***P ***were the positions before the sorting procedure in step (2). ***P***_*nr *_was transformed back to the original coordinate system by the inverse of the rotation transformation used in the PCA to obtain the noise reduced data matrix ***D***_*nr*_.

The core RSPR-NR procedure was applied to subsets composed of a relatively small number of rows randomly sampled from the large data set. The standard conditions were: the number of rows in a subset was approximately 3.33 times the number of columns in the data matrix, and the number of times each row was sampled was 20. This was done by sampling each row 20 times in different subsets, each of which was subjected to the core RSPR-NR procedure. The results of the core RSPR-NR corresponding to each row of the original data set from 20 subsets were averaged to yield the final RSPR-NR output values.

### The core idea underlying RSPR-NR

Selection of only low ranked PCs in PCA results in loss of small features. Nevertheless, a small number of PC-coordinates may be sufficient to capture even small features, provided that they are properly chosen for each row from all the PC-coordinates. This is because variances from even small features can affect determination of high ranked PCs. If this is the case, a relatively small number of PC coordinates selected for each row could capture features of various sizes quite well. This idea was explored using an Arabidopsis gene expression data set.

**Figure 1:**
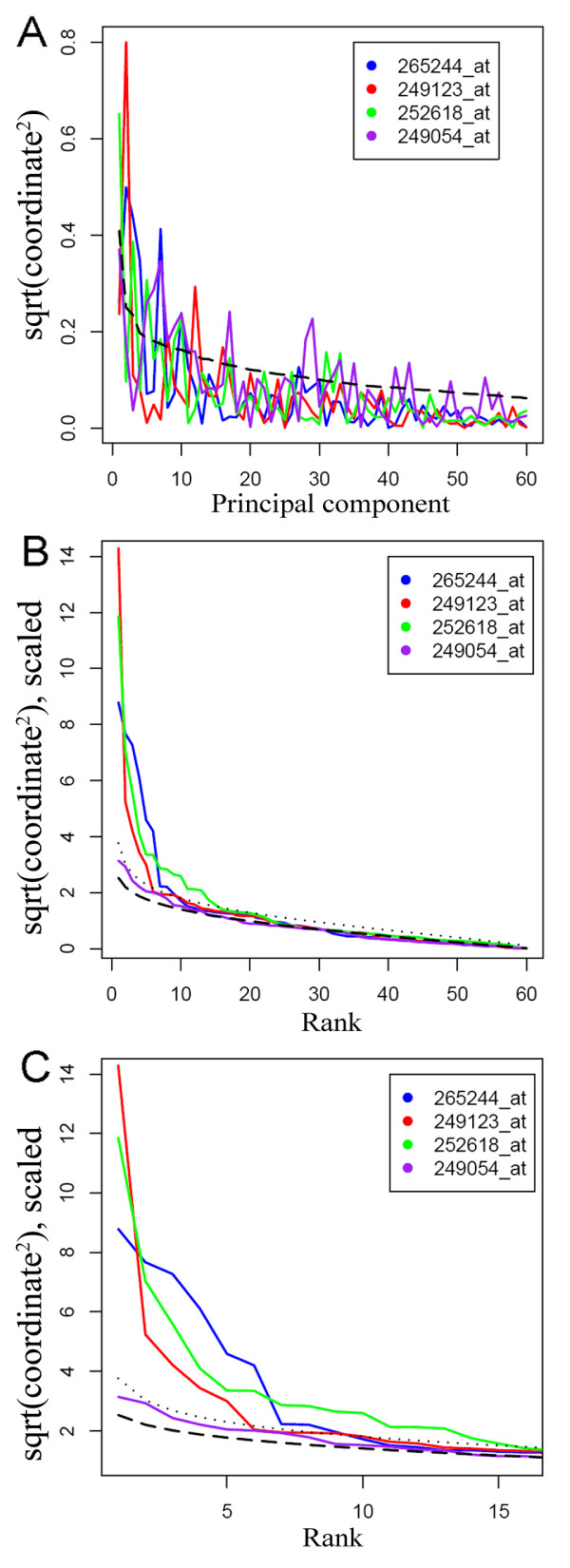
**A statistical test to determine significant PC-coordinates**. The squared PC-coordinates of four example probe sets in the PC-coordinated system are shown. The square-root scale is used for the vertical axis of each plot. (A) The squared PC-coordinates were plotted along the PCs in descending order of their associated variances. The dashed curve shows the variance associated with each PC. (B) The squared PC-coordinates were plotted in descending order of their values after scaling. The dashed curve shows the 50 percentile values of the ordered noise distributions, and the dotted curve shows a *p*-value of 0.01 (upper tail) for the ordered noise distributions. (C) A close-up view of the left part of (B). The coordinates whose squared values were above the *p*-value threshold were designated significant. Although the FDR-corrected *p*-value was used for this statistical test in the actual core RSPR-NR procedure, the uncorrected *p*-value is used for the sake of explaining the concepts in this figure.

We used log-transformed expression level ratio data because RSPR-NR assumes that the mean of the random noise distribution in each row of the data matrix is zero. In the data set used, each of the 15,863 rows corresponds to one probe set of the Affymetrix GeneChip^® ^and each of the 60 columns corresponds to one biological sample. See Methods for details of the data set. The columns of the data set were transformed to the PC-coordinates. The squared PC-coordinate values vary greatly from probe set to probe set (Figure 1A). For example, probe set 249054_at (purple) appears to have significant coordinates in some of the high ranked PCs, suggesting it may be part of a pattern not captured by the top few PCs. In step (2) of the core RSPR-NR procedure, the squared PC-coordinates were sorted in descending order for each probe set.

In step (3), the squared and ordered PC-coordinates were compared with the order distributions of squared and ordered random numbers sampled from the standard normal distribution. The latter is the random noise model. For this comparison, the squared PC-coordinates in each row were scaled so that the median value of the squared PC-coordinates in the row became equal to the median of the 50 percentile values in all the ranks of the ordered noise distributions. This scaling is based on the assumption that the squared and ordered PC-coordinates in the median and higher ranks contain only noise. In Figures 1B and 1C, the squared, ordered, and scaled PC-coordinates for four probe sets are shown as colored solid curves, and the 50 percentile values in the ordered noise distributions are shown as black dashed curves. The black dotted curves represent the *p*-value of 0.01 (upper tail) across the ranks. The squared, ordered, and scaled PC-coordinates that are larger than the dotted black curve correspond to the *p*-values smaller than 0.01 and are significant at the threshold *p*-value of 0.01. Note that in the actual RSPR-NR core procedure, the FDR-corrected *p*-value was used for the statistical test (see above). However, in this section, the fixed *p*-value of 0.01 was used as a threshold for the demonstration purpose because the FDR-corrected *p*-value depends on the *p*-value distribution and is difficult to visualize in this figure format. Using this statistical test, 9, 7, and 15 significant PC-coordinates were identified for the example probe sets 265244_at (blue), 249123_at (red), and 252618_at (green), respectively (Figure 1C). The probe set 249054_at (purple) did not have any significant coordinates despite the impression given by Figure 1A. The actual PC-coordinates selected as significant ones for 265244_at were in PCs 2, 3, 7, 4, 1, 10, 27, 11, and 14, those for 249123_at were in PCs 2, 12, 1, 8, 16, 17, and 24, and those for 252618_at were in PCs 1, 3, 5, 10, 7, 9, 31, 33, 17, 6, 22, 26, 13, 2, and 14. The orders of the listed PCs are the decreasing order of the squared PC-coordinates. The selections and orders of the listed PCs illustrate that the contributions of the PCs to the signal in different rows of the data set vary to a large extent. This observation of highly variable contributions of the PCs strongly suggests that the statistical test applied to each element of the PC-coordinated data matrix help retain small features and justifies building a noise reduction method, RSPR-NR, based on this idea.

In step (4) of the core RSPR-NR procedure, the PC-coordinates for which the corresponding squared PC-coordinates are tested insignificant in the statistical test are nullified, and the remaining PC-coordinates are transformed back to the original coordinate system to yield a noise-reduced data set.

### Simulations for Optimization and Evaluation

**Figure 2:**
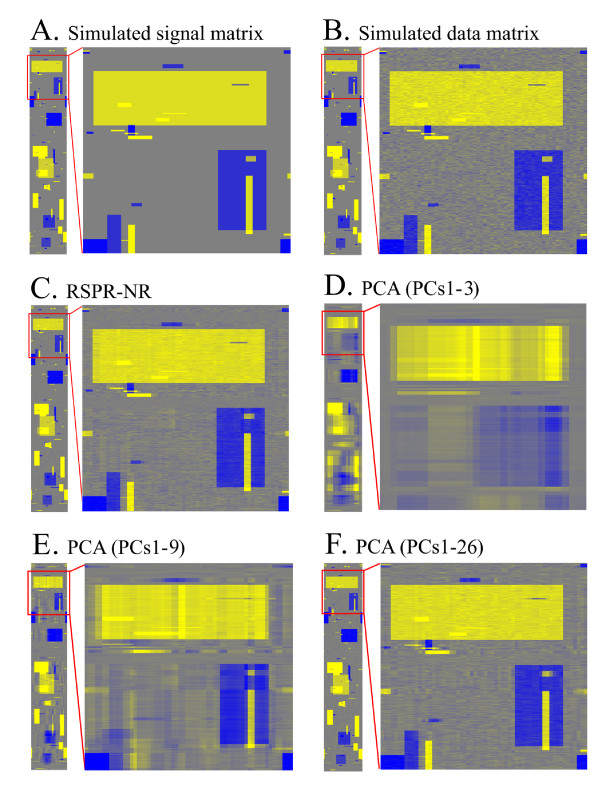
**Visualization of noise reduction performance with a simulated data set**. In each panel, the thin rectangular image on the left shows a complete view of the 2000 rows × 60 columns matrix, and the large square image on the right shows a close-up image of the red-enclosed part of the matrix. (A) Simulated signal matrix. (B) Simulated data matrix, in which random noise was added to (A). (B) was subjected to RSPR-NR (C), PCA with PCs1-3 kept (D), PCA with PCs1-9 kept (E), or PCA with PCs1-26 kept (F). See Figure 3 for the reasons that these numbers of top PCs were kept in PCA. Yellow, gray, and blue show positive, zero, and negative values, respectively. The parameters used for the simulated data set were 30 large and 60 small signal blocks and a noise variance ratio of 0.01. The parameters used in RSPR-NR were a subset row number of 200, a repeat number of 20, and an FDR of 0.0316.

It is impossible to accurately estimate the amounts of random noise and signal in real data. Therefore, for the purpose of parameter optimization and performance evaluation, we used simulated data sets in 2,000 rows × 60 columns matrices, in which we could know the exact amounts of signal and noise for each element of the data matrices. Figure 2 illustrates the steps of the simulation. First, predetermined numbers of large and small blocks were generated to mimic large and small signal features in data (30 large and 60 small blocks in Fig. 2A). This is a simulated signal matrix. Second, normally distributed random noise of a predetermined variance was added to the simulated signal matrix to obtain a simulated data matrix (Fig. 2B). The simulated data matrix was subjected to a treatment, RSPR-NR (The subset row number is 200, the repeat number is 20, and the FDR is 0.0316 in Fig. 2C; see below for these parameters.) or PCA (Figs. 2D–F). Any values in a treated matrix that differed from the original signal matrix were defined as noise. The performance of a treatment was measured by the ratio of the root mean square (RMS) of noise after the treatment to that before the treatment (noise RMS ratio). Details of the simulation procedure are provided in Methods.

**Figure 3:**
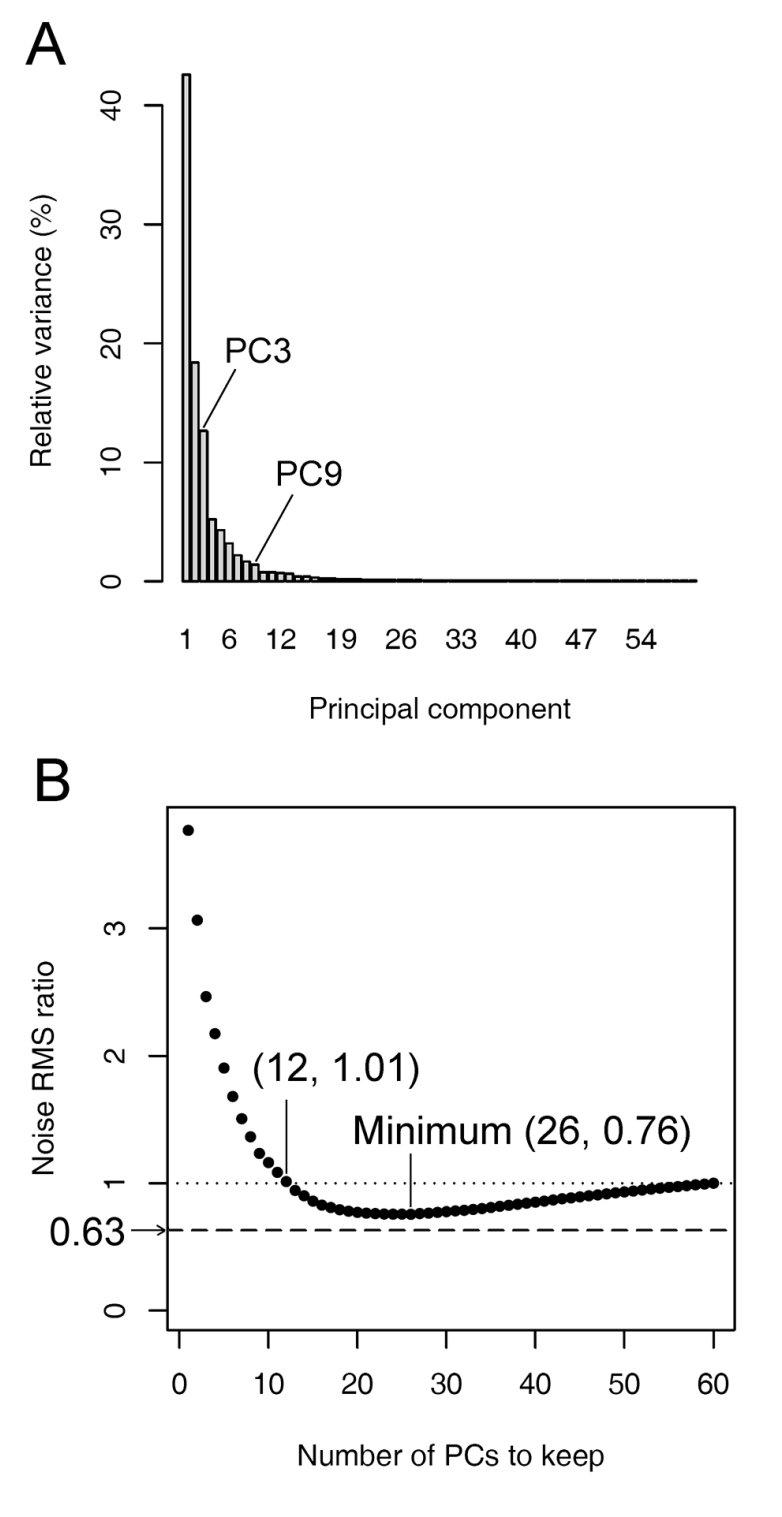
**Number of top PCs to keep in PCA for noise reduction**. The simulated data set used in Figure 2 was subjected to noise reduction by PCA. (A) The relative variance associated with each PC. The total variance of the data set was scaled to 100%. The bars for PC3 and PC9 are labeled because the variance values with the PCs immediately after them dropped noticeably. (B) The noise RMS ratio resulting from PCA with different numbers of top PCs kept. The values for two data points at PCs1-12 and PCs1-26 are indicated in the plot. The noise RMS ratio value of 0.76 at PCs1-26 was the best when PCA was used. The dashed horizontal line at the noise RMS ratio value of 0.63 indicates the noise RMS ratio value that was achieved by RSPR-NR.

It is clear that RSPR-NR retained small features very well while reducing the overall noise level (Fig. 2C). The noise RMS ratio for RSPR-NR was 0.63. To compare the performance of RSPR-NR with that of PCA, the number of top PCs that should be kept was explored. Figure 3A shows the percentage of the total variance of the simulated data matrix that can be explained by each PC. Since there were clear drops in the relative variance right after PC3 and PC9, two options, keeping the top three PCs (PCs1-3, which explain 73.6% of the total variance) and keeping the top nine PCs (PCs1-9, 91.6% of the total variance), were investigated. Unlike RSPR-NR, PCs1-3 clearly could not handle the complexity of the simulated signals (Fig. 2D). Most small features were lost or widened, and large features caused shadows of non-random noise. This tendency was still evident even with PCs1-9 (Fig. 2E). The noise RMS ratio for PCs1-3 and PCs1-9 were 2.46 and 1.23, respectively, which indicate that these treatments increase noise instead of decreasing it. Since it was not evident how many top PCs should be kept to obtain the best noise RMS ratio, we calculated the noise RMS ratio for all the possible numbers of top PCs (Fig. 3B). When the number of top PCs kept was lower than 13, PCA actually increased the noise as the noise RMS ratio is higher than 1. When the top 26 PCs were kept (PCs1-26, 97.4% total variance), the noise RMS ratio reached a minimum at 0.76, which is still larger than that of RSPR-NR, 0.63. In the visualized data matrix resulting from PCs1-26 (Fig. 2F), small features were retained well, however, the remaining noise is higher compared with RSPR-NR (Fig. 2C). Thus, using the simulated data set, RSPR-NR outperformed PCA even when the optimum number of top PCs were kept.

### Sampling Multiple Subsets for Each Row from a Large Data Set

The sensitivity of PCs for capturing small features is higher when the number of rows is limited (when the columns are the coordinates), as the variances of small features relative to that of random noise become larger. So, we decided to randomly sample rows and make subsets with a smaller row number before applying the core RSPR-NR procedure. The number of rows in each subset needs to be at least as large as the number of columns to have the number of PCs at least as large as the number of columns. On the other hand, a number of rows per subset that is too low would allow large peaks of random noise to significantly influence determination of PCs. If PCs are influenced by peaks of noise so that noise is no longer random in the PC-coordinate system, such noise will not be removed in step (4) of the core procedure. One way to reduce such an effect of noise peaks on the final output data set is to sample each row of the original data set multiple times in different subsets, and then average the results corresponding to each row of the data set from different subsets to yield a final output value for the row. Based on these considerations, we used simulation to explore two parameters: the number of rows per subset (subset row number), and the number of times each row is sampled (repeat number), to empirically determine the parameter values for good noise reduction performance.

**Figure 4:**
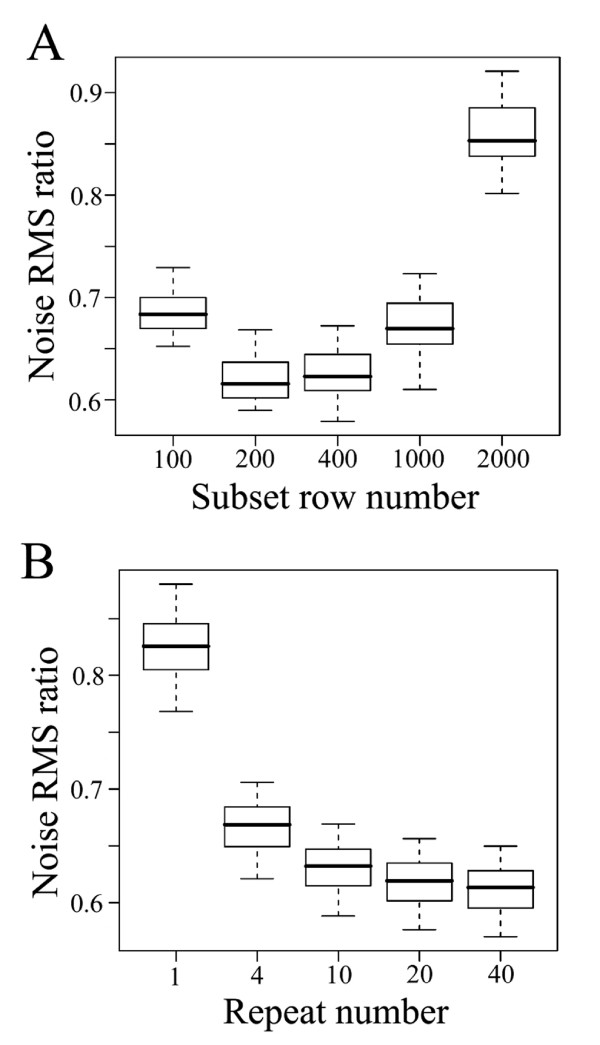
**Optimization of the subset row number (A) and the repeat number (B)**. In treatment of simulated data matrices, the effect of different subset row number and different repeat number on noise reduction was investigated. Noise RMS ratios smaller than 1 indicate noise reduction. Each box and whiskers in the box plots shows the distribution of the noise RMS ratio values from 50 simulations. The parameters used for the simulated data sets were 30 large and 60 small signal blocks and a noise variance ratio of 0.01. The FDR used was 0.0316. In (A), the repeat number was 20. In (B), the subset row number was 180.

For the sake of simplicity, only one signal block condition of 30 large and 60 small blocks, one noise variance ratio of 0.01, and one FDR of 0.0316 were used in the simulation. Figure 4A shows the noise RMS ratio distributions in 50 simulations for different subset row numbers with the repeat number fixed at 20. Note that the subset row number of 2,000 means that no subset sampling was performed as 2,000 is the number of rows in the complete data matrix. The best result was obtained using a subset row number of 200, which yielded a median RMS ratio of 0.62. However, among subset row numbers ranging from 100 to 1,000, the difference in performance was relatively small. Thus, RSPR-NR performance is relatively insensitive to the subset row number, and the choice of the subset row number is not very critical. Hereafter, a subset row number of 200, 3.33 times the number of columns in the data matrix, was used unless otherwise specified. For data sets not exactly divisible by the subset row number, the actual subset numbers were adjusted with small values to equalize the sampling numbers of each row.

A similar simulation was performed with different repeat numbers. Figure 4B shows that the higher the repeat number, the lower the noise RMS ratio, as expected. However, the level of noise RMS ratio improvement decreases as the repeat number becomes larger. A higher repeat number leads to a longer computing time. We decided that the repeat number of 20 yields satisfactory performance. The repeat number of 20 was chosen for the standard setting and used hereafter unless otherwise specified.

### Robust Performance of RSPR-NR

In the last section, only one condition of signal block numbers and one condition of noise variance ratio were used in the simulation. However, real data sets have wide ranges of signal and noise conditions, and it is generally impossible to know such conditions in real data sets. An unsupervised noise reduction method must perform well under wide ranges of conditions. We performed simulations under wide ranges of signal and noise conditions.

**Figure 5:**
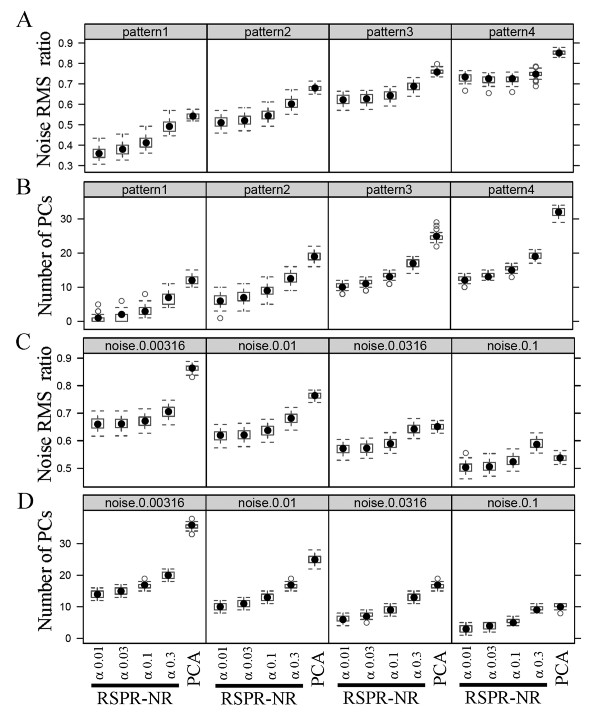
**Robust performance of RSPR-NR**. Simulated data sets with different levels of signal complexity (A) and noise variance ratio (C) were treated with RSPR-NR and PCA, and the distributions of the resulting noise RMS ratio in 40 simulated data sets are plotted. For PCA, the number of top PCs to keep was determined for the minimal noise RMS ratio in each simulation, by the procedure illustrated in Figure 3B. The distributions of the medians of the numbers of PCs kept in RSPR-NR and the distributions of the optimum number of top PCs kept in PCA for (A) and (C) are shown in (B) and (D), respectively. Four FDR conditions of 0.01, 0.0316, 0.1, and 0.316 were used in RSPR-NR and indicated as α 0.01, α 0.03, α 0.1, and α 0.3. The signal pattern conditions used in (A) and (B), which are indicated at the top of the plots, are 10 and 20 (pattern 1), 20 and 40 (pattern 2), 30 and 60 (pattern 3), and 40 and 120 (pattern 4) large and small blocks. A noise variance ratio of 0.01 was used in (A) and (B). The noise variance ratios used in (C) and (D), which are indicated at the top of the plots, are from left 0.00316, 0.01, 0.0316, and 0.1. For the signals, 30 and 60 large and small blocks (pattern 3) were used in (C) and (D). For all panels, a subset row number of 200 and a repeat number of 20 were used in RSPR-NR.

Figure 5A shows the distributions of the noise RMS ratios resulting from RSPR-NR with four different FDR values, 0.01, 0.0316, 0.1, and 0.316 (labeled as α 0.01, α 0.03, α 0.1, and α 0.3 at the bottom of Fig. 5) and PCA with the optimum number of top PCs kept when different levels of signal complexities were applied to the simulated signal matrices. Each box and whiskers shows the distribution of the noise RMS ratios in 40 simulations. The simplest pattern 1 had 10 large and 20 small signal blocks while the most complex pattern 4 had 40 large and 120 small signal blocks. The noise variance ratio was fixed at 0.01. In each signal pattern, RSPR-NR accomplished noise reduction (noise RMS ratio < 1), and the performance was relatively insensitive to the FDR value (Fig. 5A). Furthermore, at any data complexities tested, RSPR-NR with any FDR values tested outperformed PCA with the optimum number of top PCs kept. For each simulation, the median number of PC-coordinates kept per row in RSPR-NR and the optimum number of top PCs kept in PCA were recorded (Fig. 5B). Note that in RSPR-NR the PC-coordinates kept were not necessarily in the top PCs and the number of them varies row by row. The optimum number of top PCs kept in PCA increased as the pattern became more complex. Whereas the optimum number was slightly above 10 with pattern 1, the optimum number exceeded 30, which is the half of the original dimensionality, with pattern 4 (Fig. 5B, far right section). The median number of PC-coordinates kept per row in RSPR-NR also increased, indicating that RSPR-NR automatically adjusted the number of PC-coordinates kept per row according to the complexity of the signal. However, the numbers of them in RSPR-NR were always smaller than those in PCA.

Figure 5C shows the noise RMS ratios resulting from RSPR-NR and PCA when different levels of the noise variance ratio (0.00316, 0.01, 0.0316, and 0.1) were applied to the simulated data matrices. The signal pattern 3 was used, and each box and whiskers shows the distribution of the noise RMS ratios in 40 simulations. With all the noise variance ratios tested and with all the FDR tested, RSPR-NR showed substantial noise reduction. It always outperformed PCA with the optimum number of top PCs to keep, except for one condition with the highest noise variance ratio of 0.1 and with the highest FDR of 0.316. The noise reduction performance of RSPR-NR increased as the noise level gets higher. Again the performance of RSPR-NR was relatively insensitive to the FDR condition except for FDR of 0.316. The optimum number of top PCs kept in PCA decreased as the noise level increased (Fig. 5D). At the lowest noise level (noise variance ratio = 0.00316), the optimum number of top PCs kept exceeded 30. The median number of PC-coordinates kept per row in RSPR-NR also decreased as the noise level increased, indicating automatic adjustment of the number of PC-coordinates kept per row in RSPR-NR. However, again numbers of them in RSPR-NR were always smaller than those in PCA with the optimum number of top PCs to keep.

The simulations with various signal complexities and noise levels demonstrated that RSPR-NR performs well within the tested ranges, and its performance is relatively insensitive to the FDR level. This robust noise reduction performance of RSPR-NR makes it suitable for unsupervised noise reduction. In contrast, the optimum number of top PCs kept in PCA varies over a wide range dependent on the complexity of signal and the noise level. We could determine the optimum number of top PCs for PCA in the analyses shown in Figures 3 and 5 because we knew the exact signal in the simulation. In real data, we cannot determine the optimum number of top PCs in this way. The optimum number of top PCs for the best noise RMS ratio performance is much higher than the number that can be estimated by a conventional method based on the variance associated with each PC (Fig. 3). A severe underestimate of the number of top PCs kept in PCA results in an unsatisfactory noise reduction performance – it could even increase the noise RMS (Fig. 3B). Therefore, PCA is not as good an unsupervised noise reduction method as RSPR-NR.

### Noise Reduction with Actual Data

Whereas simulations allow exact quantitation of noise in a data set before and after a treatment, block signals used in the simulations may not be good approximations of signals in actual expression profile data sets. Therefore, it is important to demonstrate that RSPR-NR provides a benefit in analysis of a real expression profile data set. It is challenging to identify reasonable metrics to evaluate usefulness of RSPR-NR using a real data set. We chose to use the Gene Ontology (GO) term conservation in evaluation of RSPR-NR performance. The GO terms comprise a controlled vocabulary to describe gene and gene product attributes in three categories of molecular function, biological process, and cellular component [6]. It has been reported that genes involved in related biological processes tend to have similar expression profiles [7-10]. If this is true, we can expect reduction of random noise to result in statistical enrichment of similarly-regulated genes among genes that share GO terms.

Among the three categories of GO terms, we chose to use the biological process and the cellular component categories because the other category, molecular function, does not imply similarity in expression profiles. For example, different genes with a molecular function of "transcription factor activity" can be regulated very differently. We defined GO term relatedness as follows. To conclude that members of a gene pair share GO terms, we required the Jaccard similarity, which is the ratio of the element numbers between the intersection and the union of the GO term sets for the two genes, to be equal to or greater than 0.5. To conclude that members of a gene pair do not share GO terms, we required that they not share any GO process and component terms, and have at least 6 terms in union, thereby restricting gene pairs to very well annotated ones.

**Figure 6:**
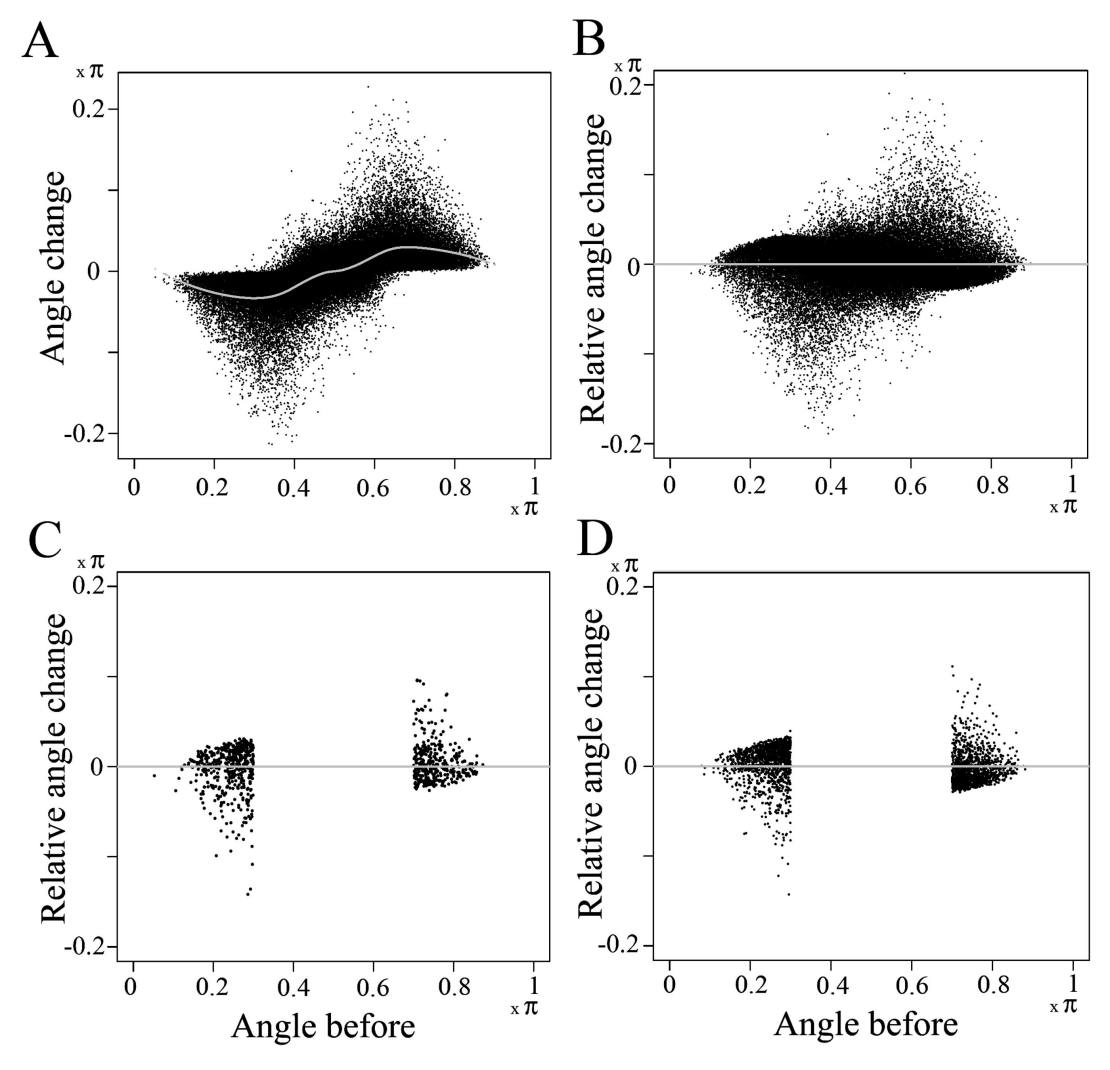
**Preferential correlation changes between gene pairs**. RSPR-NR was applied to the Arabidopsis expression profile data set. The cosine correlations between each of 80,000 randomly sampled gene pairs before and after RSPR-NR were recorded. The angle corresponding to each cosine correlation value was used in the plots. (A) The angle change from before RSPR-NR to after RSPR-NR is plotted along the angle before RSPR-NR, for the sampled gene pairs. A loess fit curve for the distribution is shown in gray. (B) From (A), the loess fit curve values were made zero (gray line) to normalize the distribution in small ranges of the angle before. The angle change value after this loess-based normalization is called the relative angle change value. This relative-angle-change-vs.-angle-before plot is used in (C) and (D). (C) The gene pairs that share GO process and component terms (Jaccard similarity = 0.5) are plotted. (D) The gene pairs that do not share any GO process and component terms and that have more than 5 GO terms in union among the sampled gene pairs are plotted. In both (C) and (D), the gene pairs with low correlation before RSPR-NR (an angle range between 0.3π and 0.7π) are excluded. The parameters used for RSPR-NR were a subset row number of 200, a repeat number of 20, and an FDR of 0.0316. The dot sizes in the plots were adjusted according to the density of dots in each plot.

The Arabidopsis expression profile data set introduced above was used. As the total number of gene pairs in the data set exceeds 1.2 × 10^8^, for practicality 80,000 gene pairs were randomly sampled and analyzed. First, overall trends of changes in the angles between pairs of gene vectors (i.e., change in the cos^-1 ^values of the cosine correlations between gene pairs; "angle change" axis) were visualized along the angle before RSPR-NR treatment ("angle before" axis; Fig. 6A). In general, RSPR-NR improves correlations between gene pairs (i.e., the angles get smaller for the gene pairs with "angle before" < 0.5 π, and larger for the gene pairs with "angle before" > 0.5 π). This was expected as RSPR-NR is a type of dimensionality reduction method. The question is whether the gene pairs that share GO terms tend to have a higher level of correlation improvement than the gene pairs that do not. To enable a comparison of such relative angle changes, we used loess [11] to define the normalization curve along the "angle before" axis (gray curve in Fig. 6A). The gene pair data were normalized by subtracting the loess fitted values from the "angle change" values (Fig. 6B). In this relative angle change plot, the data points for the gene pairs that share or do not share GO terms were identified (Figs. 6C and 6D, respectively). The gene pairs that only had small correlation before RSPR-NR (i.e., 0.3 π ≤ "angle before" ≤ 0.7 π) were excluded from this analysis because it is likely that many of these gene pairs truly do not have significant correlations. The mean values of the relative angle change for the gene pairs sharing GO terms were -0.0029 π and 0.0047π for "angle before" < 0.3 π and "angle before" > 0.7π, respectively. This result indicates that the correlations between the gene pairs sharing GO terms were statistically increased. The mean values of the relative angle change for the gene pairs not sharing GO terms were 0.0035 π and -0.0027 π for "angle before" < 0.3 π and "angle before" > 0.7 π, respectively. This result indicates that the correlations between the gene pairs not sharing GO terms were statistically reduced.

To test the significance of these observations, nine more randomly sampled sets of 80,000 gene pairs were analyzed (Table 1). The means of the mean relative angle changes for the total of 10 samples were -0.0040 π, 0.0044 π, 0.0033 π, and -0.0026 π for sharing GO terms, "angle before" < 0.3 π and > 0.7 π, and not sharing GO terms, angle before" < 0.3 π and > 0.7 π, respectively. All these sample means were significant as the probabilities of any of them being zero or having the opposite sign were < 10^-6 ^(one-sample, one-sided *t*-test). These relative angle change values may seem small. However, the mean and SEM of the means of the absolute values of the relative angle changes of all the sampled gene pairs across ten sampled sets were 0.01600 π ± 0.00002 π. So, -0.0073 π, the mean relative angle change difference between gene pairs sharing and not sharing GO terms (relative angle change difference) in the range of "angle before" < 0.3 π, represents -46% of the mean of the absolute values of the relative angle change (% angle change), a substantial change in correlation.

**Table 1:** Mean relative angle changes of the gene pairs sharing and not sharing GO terms^1)^

GO terms^2)^	Angle before	Mean relative angle change (mean ± SEM)	p-value^3)^	% angle change^4)^
Shared	0 – 0.3 π	-0.00400 π ± 0.00028 π	8.2 × 10^-8^	-25.0%
	0.7 – 1 π	0.00435 π ± 0.00032 π	1.5 × 10^-7^	27.2%
Not shared	0 – 0.3 π	0.00328 π ± 0.00022 π	4.9 × 10^-8^	20.5%
	0.7 – 1 π	-0.00257 π ± 0.00015 π	1.7 × 10^-8^	-16.0%

^1) ^A summary of the results from 10 sets of 80,000 randomly selected gene pairs. ^2) ^GO biological process and cellular compartment terms; see Fig. 5 for the exact rules for shared and not shared. ^3) ^The probability of the mean relative angle change being zero or having the opposite sign by one-sample, one-sided *t*-test. ^4) ^The ratio in percents of the mean of the mean relative angle changes of the selected gene pairs to the mean of the absolute values of the relative angle changes of all the sampled gene pairs.

**Figure 7:**
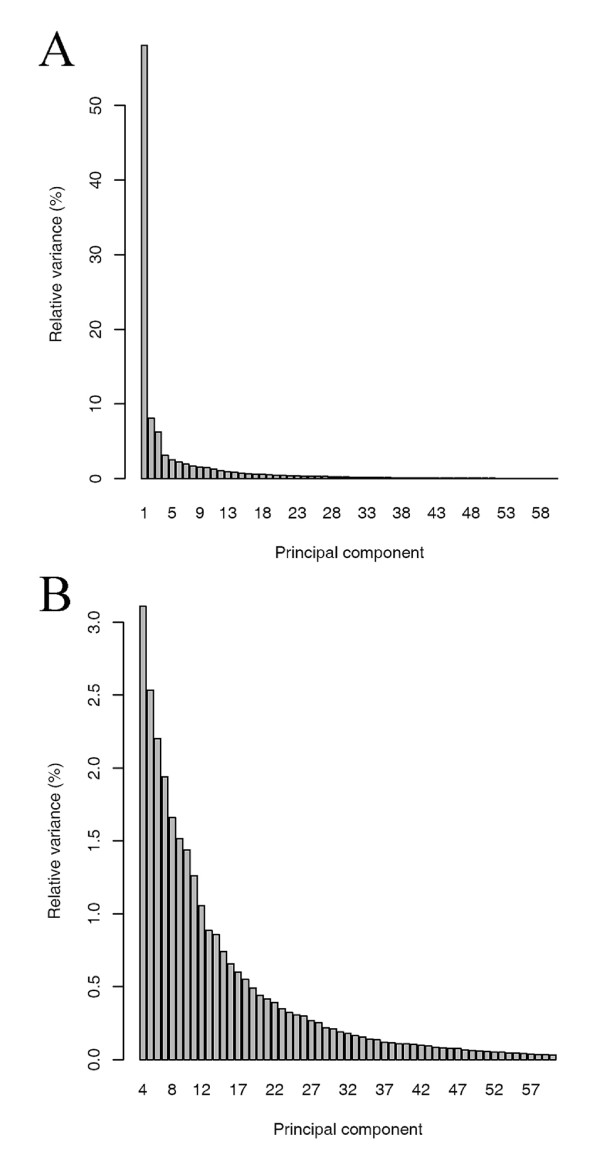
**Variance associated with each PC in PCA with the Arabidopsis expression profile data set**. (A) The variance associated with each PC (PC1 to PC60) is plotted. (B) A close up view of (A) in the range starting with PC4.

**Figure 8:**
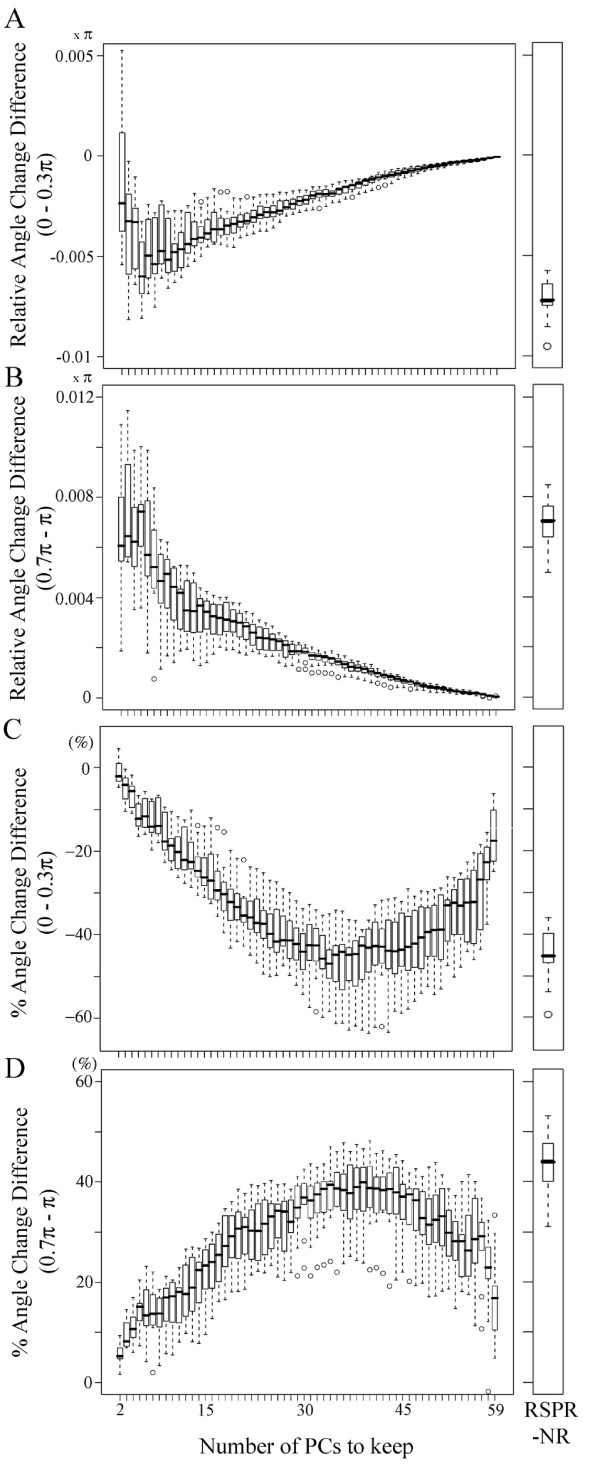
**Relative angle change difference and % angle change difference with PCA**. The distributions of the relative angle change differences (A and B) and of the % angle change differences (C and D) in ten randomly sampled sets of 80,000 gene pairs are plotted for each number of top PCs kept (2 to 59) in PCA. A positive correlation range in the angle before (< 0.3 π, A and C) and a negative correlation range in the angle before (> 0.7 π, B and D) are shown separately. In (A) and (C), the smaller the difference values are, the selectivity in preferential correlation increase with the gene pairs sharing GO terms is higher. In (B) and (D), the larger the difference values are, the selectivity is higher. In comparison, the distribution of the difference values obtained by RSPR-NR in ten randomly-sampled gene pairs is shown on the right of each panel.

The relative angle change differences and the % angle change differences, separately for the ranges of "angle before" < 0.3 π and "angle before" > 0.7 π, were also determined with the results from PCA. The relative variance explained by each PC did not provide a clear idea for a possible optimum number of top PCs to keep (Fig. 7). Therefore, the relative angle change differences and in the % angle change differences were measured for numbers of top PCs kept ranging from 2 to 59 in ten 80,000 gene pair sets, and the distributions of the difference values are shown in box plots for each number of top PCs kept (Fig. 8). For comparison, the distributions of the difference values in ten 80,000 gene pair sets resulting from RSPR-NR are also shown on the right of each panel. Figures 8A and 8B show the distributions of the mean relative change differences between the gene pairs sharing and not sharing GO terms, in the ranges of "angle before" < 0.3 π and "angle before" > 0.7 π, respectively, for each number of top PCs kept in PCA. In the range of "angle before" < 0.3 π, when PCs1-5 were kept, the median of the relative angle change difference was minimum at -0.0060 π, which means that on average the gene pairs sharing GO terms had the correlation improved by the amount corresponding to 0.0060 π, compared to the gene pairs not sharing GO terms (Fig. 8A). In the range of "angle before" > 0.7 π, in which the correlations between the gene pairs were negative before PCA, when PCs1-5 were kept, the median of the relative angle change difference was maximum at 0.0074 π (Fig. 8B). This means that on average the gene pairs sharing GO terms had the correlation improved (i.e., more negative correlation) by the amount corresponding to 0.0074 π, compared to the gene pairs not sharing GO terms. Since the medians of the relative angle change differences resulting from RSPR-NR were -0.0072 π and 0.0070 π, in the ranges of "angle before" < 0.3 π and "angle before" > 0.7 π, respectively (right panels in Figs. 8A and 8B), the improvements of the correlations between gene pairs were better than or comparable to the best cases of PCA.

When the % angle change differences were determined with the results from PCA, the optimum numbers of top PCs kept were very different from those with the relative angle change differences. In the range of "angle before" < 0.3 π (Fig. 8C), when PCs1-34 were kept, the median of the % angle change differences was minimum at -47%. In the range of "angle before" > 0.7 π, when PCs1-39 were kept, the median of the % angle change differences was maximum at 40% (Fig. 8D). The corresponding difference median values with RSPR-NR were -45% and 44%, respectively. Therefore, the performance of RSPR-NR in the % angle change difference was comparable to that of the best cases of PCA.

When PCs1-5 were kept, resulting in the best relative angle change differences, the improvements in the % angle change differences were small (-12% and 15% in the ranges of "angle before" < 0.3 π and "angle before" > 0.7 π, respectively). This indicates that when a small number of top PCs are kept, such as PCs1-5, on average the correlations between gene pairs increase substantially due to severe dimensionality reduction, regardless of whether or not GO terms are shared. Therefore, the selectivity in favoring the gene pairs sharing GO terms for correlation increase is low. Thus, with PCA it is impossible to optimize both the relative angle change difference and the % angle change difference at the same time. In contrast, RSPR-NR can achieve performance comparable to the best cases of PCA in both the relative angle change difference and the % angle change difference.

## Discussion

Using simulated data sets, we demonstrated that the noise reduction performance of RSPR-NR is robust over wide ranges of signal complexity and noise level in data sets, and the FDR used in the procedure (Fig. 5). This robustness is important for applying RSPR-NR to real data sets. Using real data, it is usually impossible to know the exact signal complexity, especially concerning small signal features, and levels of random noise. Therefore, it is impossible to select parameters for the best results. RSPR-NR can produce nearly optimal results over wide ranges of signal complexity and noise variance ratio, without adjusting the FDR parameter. In contrast, PCA could easily result in poor performance with real data because there is no definitive way to optimize the number of PCs kept for noise reduction performance using particular real data sets. To obtain an idea about noise reduction performance with a real data set, we used selective improvements of the correlation between gene pairs sharing GO terms over gene pairs not sharing GO terms. Based on this test, it became clear that in PCA, it is impossible to simultaneously optimize the number of top PCs kept for both the relative angle change difference and the % angle change difference. In contrast, RSPR-NR can achieve relative angle change difference and % angle change difference values that are comparable to or better than the values optimized separately in PCA, without parameter adjustment. So for real data sets RSPR-NR is superior to PCA for reduction of random noise.

How does RSPR-NR achieve the robust noise reduction? One mechanism is that the statistical test used in the core RSPR-NR procedure (Fig. 1) automatically adjusts the threshold level for significant signals. If the random noise level is higher, the deviation of the squared, sorted, and scaled PC-coordinates from the 50 percentile value in each rank of the noise distribution smaller because it gets a smaller scaling factor (step 3 of the core procedure; Figs. 1B, 5C, and 5D). Consequently, a smaller number of PC-coordinates will be selected as significant. If signals are more complex, the statistical test results in the opposite effect: a larger deviation of sorted squared PC-coordinates from the 50 percentile value in each rank of the noise distribution due to a larger scaling factor and a larger number of significant PC-coordinates (Figs 5A and 5B). A second mechanism is application of the core procedure to one subset at a time with each subset having a relatively small row number (Fig. 4A). This increases the probability that a small number of PCs adequately capture small features and allows RSPR-NR to accommodate complex data sets. The third mechanism is sampling of each row in multiple different subsets, and averaging of the results of the core procedure for each subset (Fig. 4B). This procedure reduces the likelihood that large peaks of noise remain in the final output data set and improves performance under a high noise condition.

It is worth emphasizing that all these mechanisms could be implemented because RSPR-NR makes a decision about a significant signal in each element of the PC-coordinated data matrix. This clearly differentiates RSPR-NR from PCA as a statistical noise reduction method. For the element-by-element decision making, the scaling factor to compare with the noise distribution is determined for each row of the PC-coordinated data matrix. This row-by-row scaling design of RSPR-NR could provide another advantage. Although we applied random noise with the same variance to the entire data matrix in the simulation, it is conceivable that the noise variance may vary in different rows, that is, there may be gene-specific variation in the random noise level in expression profile data. With this row-by-row scaling design, RSPR-NR is capable of handling such row-specific noise levels.

We applied RSPR-NR to Arabidopsis expression profile data, and the results were evaluated using an assumption of a correlation between GO term conservation and expression profile similarity among gene pairs. We demonstrated that statistically, the gene pairs that share GO process and component terms have their correlations increased while the gene pairs that do not share GO terms have their correlations decreased by both RSPR-NR and PCA (Figs. 6 and 8). This strongly suggests that RSPR-NR and PCA reduced noise from the actual expression profile data set and that this test may be generally applicable to examine the performance of a noise reduction method in expression profile data sets. However, this test method is not perfect. It is possible that the fundamental assumption of a correlation between the GO term conservation and expression profile similarity may not always apply well, and it could depend on data sets. The expression profiles used in this study were collected from experiments related to Arabidopsis responses to pathogen attack. If expression profile data collected under more diverse experimental conditions were used, the correlation between gene pairs sharing GO terms might be higher. In addition, not all GO terms used appear to be useful for this purpose. For example, the GO process terms include "developmental processes". It is easy to imagine that genes with this term have diverse expression patterns according to the specific developmental process(es) these genes are involved in. More intensive studies with various expression profile data sets are needed to better evaluate performance of RSPR-NR with real data sets.

## Conclusion

RSPR-NR is a truly unsupervised method for reduction of random noise in a large data matrix and is highly robust against variation in the signal complexity and the noise level in data. In this work, we have applied the following concepts for noise reduction: interpretation of the PCs as a mere signal-rich coordinate system; sorting the PC coordinates in the order of contribution; applying a statistical test using the noise distribution to each element of the PC-coordinated data matrix; applying the above core procedure to subsets with relatively small row numbers; averaging the results from multiple subsets for each row. All these contribute to robust performance of RSPR-NR. With more and more large and complex data sets becoming available in biology, RSPR-NR, an unsupervised statistical noise reduction method, will be a useful tool for improving data quality.

## Methods

### Expression Profile Data Set

The expression profile data used are from the set of 11 experiments we previously used [12]. They were generated using the Affymetrix ATH1 Arabidopsis genome GeneChip^® ^(22,746 probe sets) [13] and obtained from NASCArrays . After preprocessing and removal of the probe sets that are always expressed at very low levels as previously described [12], log_2_-transformed expression level ratios were calculated using the values from the appropriate control samples. The resulting log_2_-transformed expression level ratio data matrix of 15,863 probe sets (rows) and 60 biological samples (columns) was used in the work described here.

### Evaluation of Noise Reduction Performance Using Simulated Data Sets

**Figure 9:**
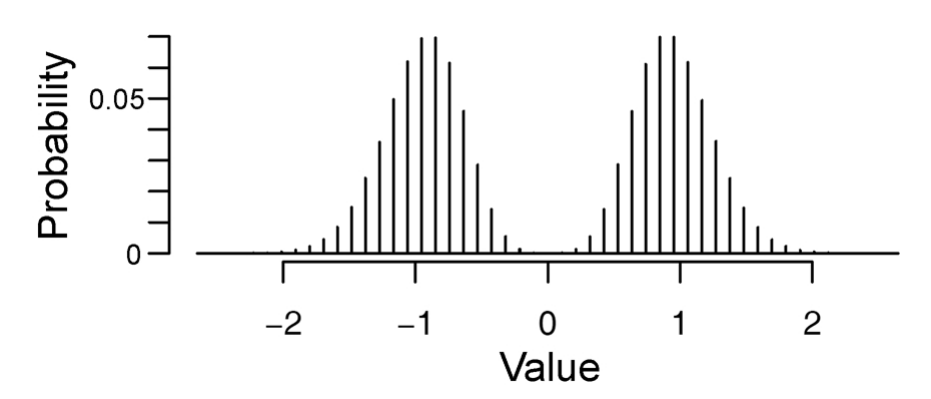
**The distribution of the population from which simulated signal values were sampled**.

Simulated data sets were used to evaluate the performance of RSPR-NR and to optimize parameters. The data matrix size used was 2,000 rows × 60 columns. The data matrix had a closed structure, as the top end of the matrix was connected to the bottom end and the left end of the matrix was connected to the right end. Two types of signal block patterns, large and small, were simulated. For the row size of a large block, a random number was sampled from a normal population with a mean of 8 and standard deviation of 3.5 (*N*(*8,3.5*^2^)), squared, and rounded. Similarly, for the column size of a large block, a random number was sampled from *N*(*2.5,2*^2^), squared, and rounded. For the row and column sizes of a small block, random numbers were sampled from *N*(*3,4*^2^) and *N*(*2,3*^2^), respectively, their absolute values were taken, and rounded. For any of the row and column numbers, if the value was larger than the data matrix size, more random numbers were sampled. The averages of the row and column sizes for large blocks were approximately 80 and 12, respectively, and those for small blocks were approximately 4 and 3, respectively. The numbers of large and small block patterns were predetermined. The value assigned for each block was randomly sampled from a bimodal discrete population symmetric with respect to zero with a probability of zero at zero (Figure 9), which was generated as follows: A Poisson population with a mean of 8, added to 1 (to make all the values positive), was scaled to a variance of 1, and duplicated to create a set with the same absolute values but a negative sign. Each signal block was placed at a random location in the data matrix. If an overlap between blocks occurred, the value for the overlapping area was the sum of the values from the overlapping blocks. A data matrix with simulated signal block patterns was designated as a signal matrix. Normally-distributed random noise of the predetermined variance relative to the signal variance in the blocks was added to each element of the signal matrix to obtain a simulated data matrix, which was subjected to PCA, RSPR-NR, or no treatment.

### Evaluation of Noise Reduction Performance Using an Actual Data Set

The Arabidopsis expression profile data set described above was subjected to RSPR-NR and PCA. The cosine correlations (also known as the uncentered Pearson correlations) between pairs of genes before and after RSPR-NR or PCA were compared. For better visualization, the angle value corresponding to the cosine correlation value was used. We randomly sampled 8 × 10^4 ^gene pairs out of more than 1.2 × 10^8 ^possible gene pairs and performed analysis with the sampled gene pairs. The angle change was defined as the angle after RSPR-NR or PCA treatment minus the angle before the treatment. The angle change was plotted against the angle before the treatment (Fig. 6A). To allow comparison of angle changes among gene pairs with similar before-treatment angle values, the loess fit [11] with the span value of 0.3 was calculated and subtracted from the angle change, resulting in the normalized plot of relative angle changes vs. before-treatment angles (Fig. 6B). Gene Ontology (GO) terms [6] were used to select groups of gene pairs that are likely to have smaller or larger angle changes. For each gene pair, the Jaccard similarity and the number of the union of their GO terms in the categories of biological process and cellular compartment were calculated. The Arabidopsis GO annotation, "ATH_GO_GOSLIM_20080301.txt", was downloaded from The Arabidopsis Information Resource (TAIR, ) [14]. For each group of gene pairs selected based on GO term characteristics, the mean relative angle change in the normalized plot was determined (relative angle change). The gene pair sampling and the analysis were repeated ten times, and the mean or median relative angle changes from the ten trials were determined.

## Abbreviations

FDR: false discovery rate; GO: Gene Ontology; PC: principal component; PCA: principal component analysis; RMS: root mean square; RSPR-NR: row-specific, sorted principal component-guided noise reduction.

## Authors' contributions

JWF developed the core RSPR-NR procedure. FK conceived of and oversaw the study and developed the subset procedure and test methods.
